# An Investigation of the Saccharides Profile and Metabolic Gene Expression in Muskrat Scented Glands in Different Secretion Seasons

**DOI:** 10.3390/ani14243705

**Published:** 2024-12-22

**Authors:** Juntong Zhou, Defu Hu, Nuannuan Feng, Shuqiang Liu, Junqing Li

**Affiliations:** Department of Ecology, School of Ecology and Nature Conservation, Beijing Forestry University, Beijing 100083, China; zhoujuntong908020@126.com (J.Z.); fnn1990123@163.com (N.F.); shuqiangliu@163.com (S.L.); lijq@bjfu.edu.cn (J.L.)

**Keywords:** muskrat scented gland, saccharides, LC-MS, transcriptome analysis, secretion season

## Abstract

The adult male muskrat has a pair of scented glands, which show clear seasonal changes in their developmental status between the secretion season and non-secretion season. During the secretion season, the scented glands are much larger than in the non-secretion season, with the metabolism of glandular cells increasing and a large amount of musk being produced. In this work, the blood, musk, and scented gland tissue were collected from three healthy adult male muskrats during secretion season (September). And the blood and scented gland tissue from another three healthy adult male muskrats during the non-secretion season (November) were also sampled. The saccharides from blood and musk were detected by liquid chromatography–mass spectrometry (LC-MS), indicating the saccharides are concentrated in the scented glands during the secretion season. What is more, transcriptome analysis was employed to investigate the expression patterns of saccharides’ pathways, suggesting some saccharides’ metabolism-related genes undergo significant seasonal changes. Above all, scented gland saccharides’ metabolism displays seasonal differences, and the enhancement in saccharides’ metabolic activity during the secretion phase maintains glandular proliferation and secretion function.

## Simple Summary

The purpose of this study was to investigate the saccharides profile in muskrat scented glands in different secretion seasons. The significant changes in the metabolic pathways of nine saccharide substances in the scented gland were detected. The regulatory changes in saccharides metabolism in the scented gland ensure its metabolism and energy requirements during the secretion season. These results will help elucidate the regulatory mechanism of the gland during the musk secretion season.

## 1. Introduction

As one of the economically significant animals, the muskrat (*Ondatra zibethicus*) is famous for its musk secretion and fur. Adult muskrats can develop a pair of secretory glands located between the abdominal muscles and skin near the tail [1]. The scented glands are composed of glandular cells, supporting cells, and excretory ducts. During the secretion season, the scented gland is full of musk and its transverse diameter can reach 15~20 mm, and the length can reach to about 37 mm. In contrast, the scented glands gradually become soft and smaller during the non-secretion season, and the acini are finally replaced by connective tissue [2,3]. The milky white oily liquid muskrat musk is a precious animal fragrance known as American musk; its main components are the same as natural musk, including muskone [4], nor-muskone, etc. The distinctive scent of musk is attributed to the abundance of a specific ketone, muscone, which is produced by the musk gland [5,6,7]. Although the anti-hypoxia effect of muskrat musk is not as good as that of natural musk, the anti-inflammatory activities of muskrat musk and natural musk are similar [8,9]. Therefore, the muskrat musk has important medicinal and economic value in the fields of medicine and the daily activities of the chemical industry [10].

As one of seasonal breeding animals with sexual activity lasting from March to October (reproductive period or secretion season), male and female muskrat mate, conceive, give birth, and secrete musk during this period. Compared with the secretion period, the reproductive hiatus period (non-secretion season) is from November to February of the following year; during this period, muskrats do not exhibit significant reproductive behavior and secretion musk [11].

Saccharides are a natural polymer [12] and basic substance for maintaining physical health [13], which have important structural and nutritional functions for animals and plants. Saccharides and their compounds are mainly distributed in natural animals, plants, microorganisms, and fungi [14]. They are involved in maintaining the normal functions of body cells and have many physiological functions [15], such as anti-tumor [16,17], anti-inflammatory, antioxidant [18,19], and immune regulation [20]. Although saccharides play an extremely important role in metabolism, there are few reports on the distribution and function of saccharides in muskrat secretion glands. Interestingly, in the study of musk deer, Xu [21] proposed that carbohydrates, amino acids, and fatty acids were enriched in the secreted glands at three secretion periods.

Currently, there is very little research on the distribution and metabolism of saccharides in the seasonal changes in scented glands. In this work, for the first time, the saccharide profile of muskrat blood and musk liquid was analyzed by metabolomics; furthermore, transcriptome analysis was employed to study the expression of genes related to saccharide metabolism and evaluate the saccharide metabolism in scented glands.

## 2. Materials and Methods

### 2.1. Animals and Sample Collection

The experimental muskrats used in this work were purchased from Jinmu Technologies (Xinji City, Hebei Province, China). All the muskrats used in the experiment were captive in the flat test enclosure and had no contact with the dam. The daily diet of captive muskrats is managed by the farm, with vegetables, fruits, grains, and bean seeds. Six healthy adult male muskrats of a similar age (between 1 and 1.5 year), size, and weight were selected; three of them were obtained in September (the secretion season) and another three were obtained in November (the non-secretion season). The blood samples were collected after the muskrats were treated with isoflurane for general anesthesia. The collected blood samples were pipetted to tubes at 37 °C stewing for 1 h, then centrifuged at 3000 rpm for stratification. The supernatant was centrifuged at 4 °C at 12,000 rpm for 10 min again. After that, 0.2 mL of serum was pipetted in a 1.5 mL tube and stored at −80 °C in a freezer for subsequent metabolome detection.

After the blood sample collection, the muskrat cavity was opened and the scented glands were found. The scented glands during two periods were collected and their sizes measured. Then, the liquid musk was extracted from the scented glands by syringe and pipetted into centrifuge tubes and stored at −80 °C in a freezer for subsequent metabolome detection. The scented glands tissue was cut into small pieces, packed in centrifuge tubes, and cryopreserved in liquid nitrogen for later transcriptome sequencing.

All animals were treated in accordance with the National Animal Welfare Legislation. All experimental procedures were carried out in accordance with the guidelines on animal care established by Beijing Forestry University; the latter also approved the study.

### 2.2. Metabolome Analysis

Blood serum samples were thawed on ice and whirled for 10 s to mix well; then, 300 µL of pure methanol was added to 50 µL of plasma/serum, the mixture was whirled for 3 min, then centrifuged at 12,000 rpm at 4 °C for 10 min. Then, the supernatant was collected and centrifuged at 12,000 rpm at 4 °C for another 5 min. It was left in a refrigerator at −20 °C for 30 min, centrifuged at 12,000 r/min at 4 °C for 3 min, and 150 µL of supernatant in the liner of the corresponding injection bottle was taken for on-board analysis.

The blood sample extracts and the musk samples were analyzed using an LC-ESI-MS/MS system (UPLC, ExionLC AD, https://sciex.com.cn/, accessed on 19 March 2021); MS, QTRAP^®^ System (Sciex, Shanghai, China), https://sciex.com/, accessed on 19 March 2021)). The analytical conditions recommended by the manufacturer were as follows: UPLC: column, Waters ACQUITY UPLC HSS T3 C18 (1.8 µm, 2.1 mm × 100 mm); column temperature, 40 °C; flow rate, 0.4 mL/min; injection volume, 2 μL; solvent system, water (0.1% formic acid)/acetonitrile (0.1% formic acid); and gradient program: 95:5 *v*/*v* at 0 min, 10:90 *v*/*v* at 10.0 min, 10:90 *v*/*v* at 11.0 min, 95:5 *v*/*v* at 11.1 min, and 95:5 *v*/*v* at 14.0 min.

LIT and triple quadrupole (QQQ) scans were acquired on a triple quadrupole linear ion trap mass spectrometer (QTRAP), QTRAP^®^ LC-MS/MS system (Sciex, Shanghai, China), equipped with an ESI turbo ion spray interface, operating in positive and negative ion mode and controlled by Analyst 1.6.3 software (Sciex, Shanghai, China). The ESI source operation parameters were as follows: source temperature of 500 °C; ion spray voltage (IS) at 5500 V (positive) and −4500 V (negative); ion source gas I (GSI), gas II (GSII), and curtain gas (CUR) were set to 55, 60, and 25.0 psi, respectively; and the collision gas (CAD) was high. Instrument tuning and mass calibration were performed as SCIEX recommended with 10 and 100 μmol/L polypropylene glycol solutions in QQQ and LIT modes, respectively. A specific set of MRM transitions was monitored for each period according to the metabolites eluted within this period.

### 2.3. Transcriptome Analysis

Total RNA from each muskrat scented gland sample was isolated using TRIzol reagent (Qiagen, Valencia, CA, USA). The quantity and integrity of total RNA was assessed using an Agilent Bioanalyzer 2100 system (Agilent Technologies, San Diego, CA, USA) and 1% agarose gel electrophoresis; RNA concentration was measured using the Qubit RNA Assay Kit on a Qubit 2.0 Flurometer (Life Technologies, Carlsbad, CA, USA). The purity, concentration, and integrity of RNA samples were tested using advanced molecular biology equipment to ensure the use of qualified samples for transcriptome sequencing. Approximately 30 μg of total RNA from three individuals in each season (September and November) was used for Illumina sequencing by Biomarker technologies (Beijing, China).

Poly (A)+ RNA was enriched and purified using oligo (dT) magnetic beads and then broken into short fragments. Using these cleaved mRNA fragments as templates, first, strand cDNA was synthesized using random hexamer primer and M-MuLV reverse transcriptase. Second strand cDNA synthesis was subsequently performed using DNA Polymerase I and RNase H. Remaining overhangs were converted into blunt ends via exonuclease/polymerase activities. After the adenylation of the 3′ ends of DNA fragments, an NEBNext adaptor with a hairpin loop structure was ligated to prepare for hybridization. In order to select cDNA fragments of preferentially 240 bp in length, the library fragments were purified with an AMPure XP system (Beckman Coulter, Beverly, MA, USA). Then, 3 μL User Enzyme (NEB, Ipswich, MA, USA) was used with size-selected, adaptor-ligated cDNA at 37 °C for 15 min followed by 5 min at 95 °C before PCR. Then, PCR was performed with Phusion High-Fidelity DNA polymerase, universal PCR primers, and an index (X) primer. At last, PCR products were purified (AMPure XP system) and library quality was assessed on the Agilent Bioanalyzer 2100 system (Agilent Technologies (China) Co., Ltd., Beijing, China). The clustering of the index-coded samples was performed on a cBot Cluster Generation System using TruSeq PE Cluster Kit v3-cBot-HS (Illumia, Inc., San Diego, CA, USA) according to the manufacturer’s instructions. After cluster generation, the library preparations were sequenced on an Illumina Hiseq 2000 platform (Illumia, Inc., San Diego, CA, USA) and paired-end reads were generated.

Raw data (raw reads) of fast format were first processed in-house per scripts. In this step, clean data (clean reads) were obtained by removing reads containing the adapter, reads containing ploy-N, and low-quality reads from raw data. At the same time, Q20, Q30, GC-content, and the sequence duplication level of the clean data were calculated. All the downstream analyses were based on clean data with high quality. With the absence of a genome, sequenced reads were assembled using Trinity software (Broad Institute of MIT and Harvard, https://pmc.ncbi.nlm.nih.gov/articales/PMC3571712/pdf/nihms292662, accessed on 19 March 2021) with min_kmer_cov set to 2 by default and all other parameters also set to default.

Unigenes were annotated using BLAST (Basic Local Alignment Search Tool) searches against the NR (NCBI non-redundant protein sequences), Pfam (protein family), KOG/COG/egg NOG (Clusters of Orthologous Groups of proteins), Swiss-Prot (a manually annotated and reviewed protein sequence database), KEGG (Kyoto Encyclopedia of Genes and Genomes), and GO (Gene Ontology). Sample reads were compared with the transcript using Bowtie software (https://www.bowtiepro.com/software/, accessed on 19 March 2021). The gene expression levels were estimated by RSEM for each sample. The result was presented in terms of fragments per kilobase of transcript per million mapped reads (FPKM).

### 2.4. Statistical Analysis

For better visualization and subsequent analysis, the orthogonal projections to latent structures–discriminant analysis (OPLS-DA) method was utilized to assess the difference in saccharide metabolites in the secretion season and non-secretion season. The three muskrats in the secretion season were named MXQ1, MXQ2, and MXQ3, and the other three muskrats in the non-secretion season were named FMXQ1, FMXQ2, and FMXQ3.

Results are presented as means+ standard error of the mean (SEM) or standard deviation (SD). Student’s *t*-test was used for data analysis. A *p*-value < 0.05 was considered statistically significant. Genes with an adjusted *p*-value < 0.05 found by DESeq were assigned as differentially expressed. In the selection of differentially expressed genes, a false discovery rate (FDR) < 0.01 and a fold change ≥ 2 were employed as the standard. The resulting *p* values were adjusted using Benjamini and Hochberg’s approach for controlling the false discovery rate.

## 3. Results

### 3.1. Metabolomics Analysis of Saccharides in Muskrat Blood Serum and Musk

**Figure 1:**
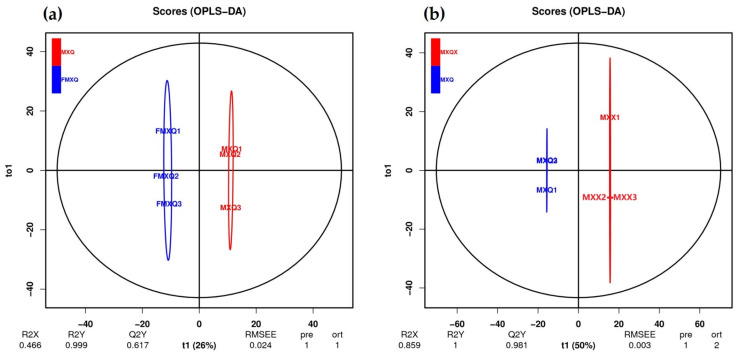
The result of OPLS-DA. (**a**) The differential analysis of saccharide metabolism in serum during the secretion and non-secretion season. (**b**) The differential analysis of saccharide metabolites in the blood serum and musk during the secretion and season. (The horizontal axis represents the score Tp of the main components in the OSC process. The vertical axis represents the score values TOR2X and R2Y of the orthogonal components in the OSC process, respectively, indicating the explanatory power of the constructed model on the X and Y matrices. The X matrix is the model input, i.e., the metabolite quantification matrix, the Y matrix is the model output, i.e., the sample grouping matrix, and Q2 represents the predictive ability of the model, i.e., whether the constructed model can distinguish the correct sample grouping based on metabolic expression levels. When Q2 > 0.5, it can be considered an effective model).

The results of OPLS-DA are displayed in Figure 1, the inter-group differences in saccharide metabolites are very significant (Figure 1a), and there are also differences between samples within the group. During the secretion season, the saccharide metabolites in the blood serum and musk show significant differences (Figure 1b).

**Figure 2:**
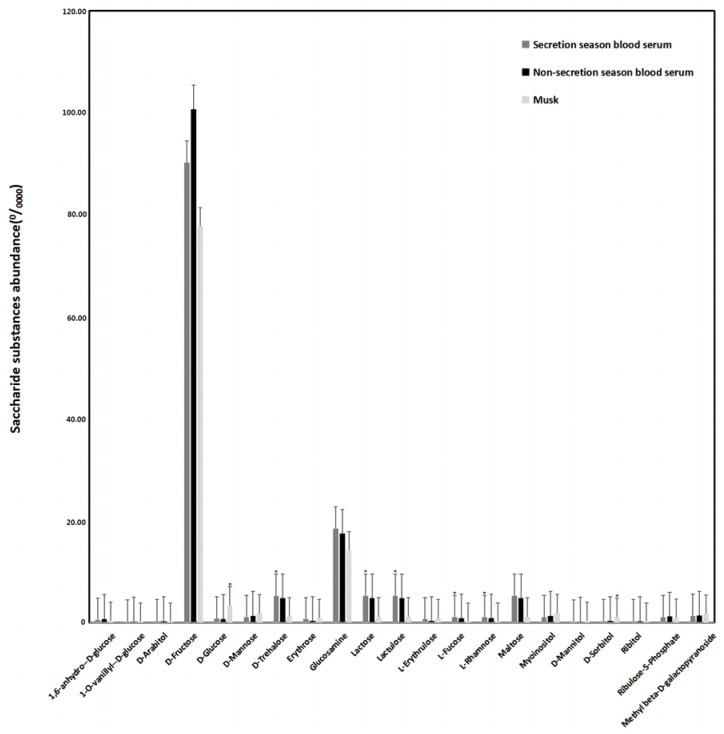
The abundance of saccharide substances (saccharides with significant changes are marked by ‘*’).

The analysis of metabolomic sequencing results reveals the expression levels of saccharide substances during both the secretion season and non-secretion season, as presented in Table 1. A total of 21 saccharide substances were detected and compared to the secretion season and non-secretion season; the expression levels of these substances remained relatively stable. The expression levels of saccharide substances in blood serum and musk during the secretion season are displayed in Table 2. As shown in Figure 2 and Table 3, a total of 21 types of saccharide substances were identified. Among them, a comparison between blood serum and musk samples revealed that the abundance of D-glucose (MEDN0220) and D-Sorbitol (MEDN0213) in musk was significantly higher than that in blood serum. Conversely, the abundance of D-trehalose (MEDN0224), lactose (MEDN0229), Lactulose (MEDN0230), L-fucose (MEDN0231), and L-rhamnose (MEDN0232) in perfumes was significantly lower than that in blood serum. The expression levels of the remaining 14 substances remained stable.

### 3.2. Transcriptome Analysis of Saccharide Metabolism-Related Genes in Muskrat Scented Gland Tissues

Transcriptome analysis of muskrat scented gland tissues collected during the secretion and non-secretion season was performed. The analysis identified nine upregulated and 20 downregulated genes. Except for the pentose phosphate pathway (ko00030) and pentose and gluconate interconversion (ko00040) signaling pathways, genes from other pathways were mainly downregulated. The most relevant changes to the expression of genes involved in saccharide metabolic and signaling pathways are listed in Table 4.

**Figure 3:**
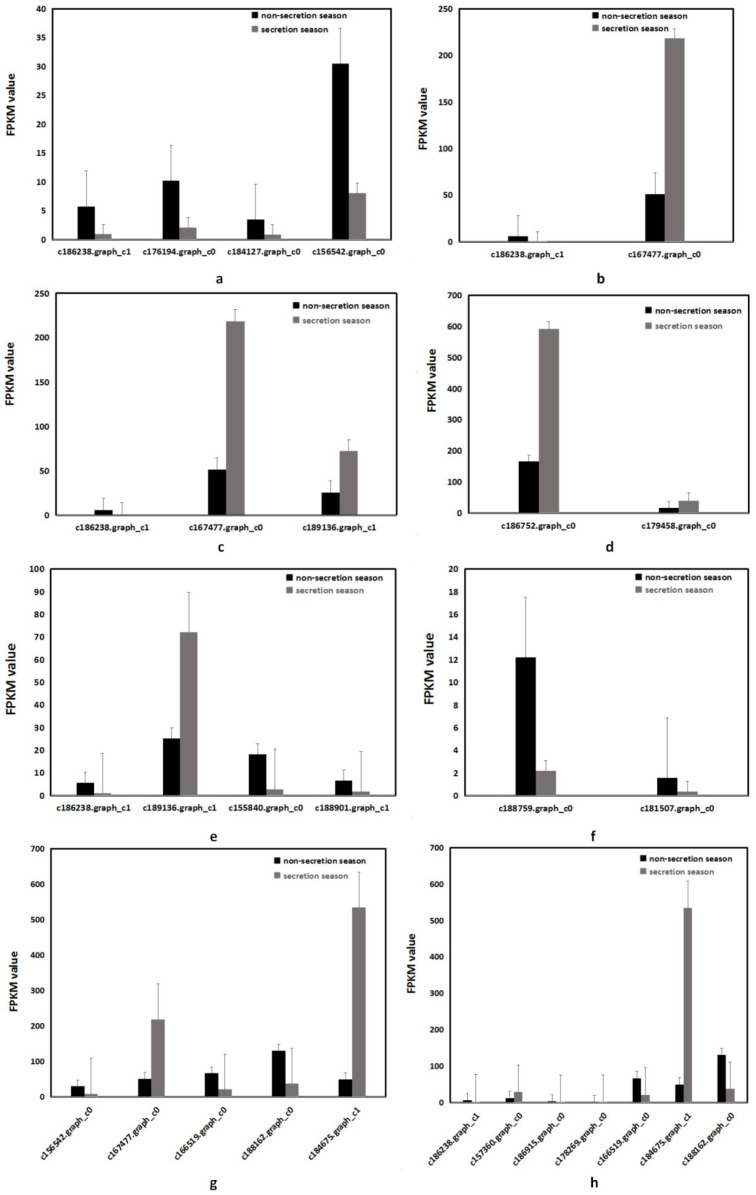
The gene expression of the transcriptome related to saccharide metabolism. (**a**) Starch and sucrose metabolism (ko00050); (**b**) galactose metabolism (ko00052); (**c**) fructose and mannose metabolism (ko00051); (**d**) pentose phosphate pathway (ko00030); (**e**) amino sugar and nucleotide sugar metabolism (ko00520); (**f**) glycosaminoglycan degradation (ko00531); (**g**) pentose and glucuronate interconversions (ko00040); (**h**) glycolysis/gluconeogenesis (ko00010). Data are presented as means + SD (*n* = 9). FDR < 0.01 and fold change ≥ 2. FDR, false discovery rate. FPKM, fragments per kilobase of transcript per million mapped reads.

The accumulation of saccharide metabolism-related transcripts involved in musk production during the secretion and non-secretion season is detailed in Figure 3. The false discovery rate for all genes was less than 0.01 (FRD < 0.01).

By comparing the gene expression profiles of samples in different seasons, differentially expressed genes were identified and then mapped to the Kyoto Encyclopedia of Genes and Genomes (KEGG) database (http://www.genome.ad.jp/kegg/, accessed on 19 March 2021) reference pathways, and this narrowed our attention to several crucial saccharide metabolic and signaling pathways. A total of 29 unit transcripts were assigned to eight pathways, namely starch and sucrose metabolism (ko00500) (Figure S1); galactose metabolism (ko00052) (Figure S2); fructose and mannose metabolism (ko00051) (Figure S3); the pentose phosphate pathway (ko00030) (Figure S4); amino sugar and nucleotide sugar metabolism (ko00520) (Figure S5); glycosaminoglycan degradation (ko00531) (Figure S6); pentose and gluconate interconversions (ko00040) (Figure S7); and glycolysis/glyconeogenesis (ko00010) (Figure S8). The red frame in the figures indicates that gene expression was upregulated during the secretion season, the green frame indicates that gene expression was downregulated during the secretion season, and the blue frame denotes genes whose expression was both upregulated and downregulated.

## 4. Discussion

The scented glands of muskrats can secrete musk, and their growth undergoes prominent cyclical changes. Here, metabolomics was utilized to analyze the saccharide profile of muskrat blood serum and scented glands for the first time, and then transcriptome analysis was employed to study the expression of genes related to saccharide metabolism in scented glands. Based on the results, it was found that there were no significant changes in the concentration of saccharides in the blood serum of muskrats between the secretion season and non-secretion season. However, it cannot be determined that saccharides will not show short-term fluctuations in the blood serum throughout the secretion season and non-secretion season. If the concentration of saccharides in the blood serum increases sharply with increased metabolism, it might affect other muskrat organs. Therefore, maintaining a stable concentration of saccharides in the blood serum throughout the entire secretion and non-secretion periods can ensure the physiological balance of the muskrat body.

According to the above results, the abundance of D-glucose (MEDN0220) and D-bitol (MEDN0213) in musk is significantly higher than that in blood serum, but the abundance of D-trehalose (MEDN0224), Lactulose (MEDN0230), lactose (MEDN0229), L-fucose (MEDN0231), and L-rhamnose (MEDN0232) in musk is lower than that in blood serum. And the expression level of the remaining 14 saccharide substances shows a stable unchanged trend. These indicate that saccharides can be enriched in the muskrat scented glands, which is consistent with early research on saccharides in the mammary glands and blood serum of cows [22]. It can be speculated that the process of accumulating saccharides in the muskrat scented glands is traced and slow, thus ensuring the physiological stability of the body. The local accumulation of saccharides can maintain the needs of individual organs during special periods without affecting the overall physiological balance.

As an important metabolic pathway, the pentose phosphate pathway (PPP, ko00030) is closely related to the occurrence and development of cell proliferation. As a key rate-limiting enzyme, glucose-6-phosphate dehydrogenase can exist as an inactive monomer and an active dimer and even as a more advanced tetramer complex, as reported [23]. And NADP+/NADPH is one of the main activity regulators of this enzyme; NADPH is responsible for regulating the activity of G6PD, and NADP+ is necessary for enzyme activity and its correct conformation [24]. During cell proliferation, G6PD is highly expressed and exhibits high enzyme activity [25]. As has been reported on HCT116 tumor cells, P53 inhibits the entire PPP pathway by directly binding G6PD to inhibit its enzyme activity. Therefore, this inhibitory effect disappears in tumors with P53 deficiency or inactivation, leading to an enhancement of the pentose phosphate pathway in the tumor and promoting intracellular biosynthesis [26].

After analyzing the gene transcriptome of scented gland tissue, it was found that the pentose phosphate pathway (PPP, ko00030) was upregulated, in which the c186752.graph_c0 (1.1.1.44/*PGD*) and c179458.graph_c0 (5.3.1.6/*rpiA*) genes regulate D-ribulose and D-ribose. D-ribose is one kind of functional pentose, with strong water solubility and a sweet taste, and it exists in all living cells. D-ribose is also an important component of RNA, some coenzymes, water-soluble vitamins, and adenosine triphosphate (ATP). As the intermediate and key component of DNA, RNA, and other nucleic acids, D-ribose occupies a core position in fat metabolism, protein, and nucleoside substances. And D-ribose is closely related to the regeneration of ATP, which plays an important role in energy metabolism and restoring physiological functions. The content of D-ribulose and D-ribose increased with the upregulation of the expression of c186752.graph_c0 (1.1.1.44/*PGD*) and c179458.graph_c0 (5.3.1.6/*rpiA*) genes. It means that the upregulation of glucose metabolism-related genes in scented gland tissues during the secretion season leads to accelerated glucose metabolism. And during this season, the scented gland tissue cells proliferate and secrete musk, which consumes a large amount of energy during cell division, proliferation, and secretion. Glucose is the main energy source of the body, providing raw materials for the synthesis of various nucleotides and nucleic acids in the body.

In the metabolism of starch and sucrose, c186238.graph_c1 (2.7.1.1/*HK*), c176194.graph_c0 (2.4.1.18/*GBE1*), c184127.graph_c0 (2.4.1.1/*PYG*), and c156542.graph_c0 (2.4.1.17/*UGT*) were downregulated genes, whose main function is to participate in glycogen breakdown, such as the c184127.graph_c0 (2.4.1.1/*PYG*) [27] gene downregulated by 1.826 times; this gene encodes a homodimer protein that catalyzed the cleavage of alpha-1,4-glucoside chains and releases glucose 1-phosphate from liver glycogen.

The C176194. graph_c0 (2.4.1.18/*GBE1*) [28] gene was downregulated by 1.826 times, which is a glycogen-branching enzyme used to catalyze and transfer the alpha-1,4-linked glucosyl units at the outer end of the glycogen chain to the alpha-1,6 position on the same or adjacent glycogen chain. The branching of glycogen chains is necessary to increase the dissolution of glycogen molecules. And the c186238.graph_c1(2.7.1.1/*HK*) gene also exists in galactose metabolism (ko00052), fructose and mannose metabolism (ko00051), and amino sugar and nucleotide sugar metabolism (ko00520). Glycolysis/glyconeogenesis (ko00010) was downregulated by 2.487 times. It encodes a glycolysis enzyme, which is expressed in all mature tissues except the pancreas and liver to promote glucose metabolism. However, it is low or even not expressed in cancer cells. Cancer cells increase their own *HK1* level by hijacking the *IEV HK1* secreted by HSCs, and then increase the level of glycolysis in cancer cells to promote cancer cell proliferation.

UDP-glycosyltransferase (UGT), one kind of protease superfamily related to glycosylation, was downregulated by 1.834 times. Landerer [29] showed that the UGT1A enzyme in the human body acts as an indirect antioxidant, catalyzing the elimination of active metabolites and playing a protective and antioxidant role in the liver. Downregulated genes can promote the proliferation of muskrat scented gland cells and the synthesis of musk during the secretion season.

In the metabolism (ko00052) of galactose, c186238.graph_c1(2.7.1.1/*HK*) was downregulated and c167477.graph_c0(1.1.1.21/*AKR1B*) was upregulated. *AKR1B* was also present in fructose and mannose metabolism (ko00051) and pentose and gluconate conversions (ko00040) were upregulated 2.293 times. That is, an aldehyde ketone reductase superfamily and a soluble monomer oxidoreductase in the cytoplasm that depends on reduced nicotinamide adenine dinucleotide phosphate (NADPH) [30], in which AKR1B1 is mainly involved in three metabolic pathways, namely ① the sorbitol synthesis pathway, where AKR1B1 catalyzes glucose to produce sorbitol, and excessive sorbitol aggregation will promote the occurrence of complications of diabetes; ② the PGF2α generation pathway, where AKR1B1 can catalyze prostaglandin H2 (PGH2) to produce prostaglandin F2α(PGF2α); and ③ the detoxification process of lipid peroxide [30,31,32]. The upregulation of the AKR1B1 gene can not only catalyze the breakdown of glucose but also promote the growth and proliferation of muskrat scented gland cells and the synthesis of musk during the secretion season.

In the metabolism of amino sugar and nucleotide sugar (ko00520), the genes c155840.graph_c0(3.2.1.14/*E3.2.1.14*), c186238.graph_c1 (2.7.1.1/*HK*), and c188901.graph_c1 (1.14.18.2/*CMAH*) was downregulated. *CMAH* was downregulated by 1.675 times, which is an enzyme that converts N-glycolylneuraminic acid (Neu5Gc) into N-acetylneuraminic acid (Neu5Ac) [33]. Sialic acid Neu5Gc is widely distributed in red meat, which cannot be digested in the human body but is deposited on the surface of organs such as the heart [34]. Some studies have pointed out that excessive intake of red meat will significantly increase the risk of cancer, cardiovascular disease, and total mortality [35]. The downregulation of *CMAH* will reduce the conversion of Neu5Gc into Neu5Ac, which is conducive to the growth and proliferation of muskrat scented gland cells and the synthesis of musk during the secretion season. The gene C189136.graph_ C1 (5.4.2.8/*manB*) also exists in the metabolism of amino sugar and nucleotide sugar (ko00520) pathway, which was upregulated by 1.677 times. The phosphomannomutase (man B) gene encodes phosphomannomutase [36]; during the glycometabolism process, phosphate−6−mannose can be converted into phosphate−1−mannose under the action of phosphomannase. It is closely related to the primary metabolic process of cells and the synthesis process of the secondary metabolite [37,38] so as to facilitate the secretion of musk liquid during the secretion period.

In the interconversion (ko00040) between pentose and gluconate, the gene c167477.graph_c0(1.1.1.21/*AKR1B*) was upregulated and the genes c156542.graph_c0 (2.4.1.17/*UGT*), c166519.graph_c0 (1.2.1.3/*ALDH2*), and c188162.graph_c0 (1.2.1.3/*ALDH1*) were downregulated. The genes 166519.graph_c0 (1.2.1.3/*ALDH2*) and c188162.graph_c0 (1.2.1.3/ALDH1) were also presented in glycolysis/glyconeogenesis (ko00010) and downregulated 1.51 and 1.632 times, respectively. The acetaldehyde (ALDH) encoded by them is an enzyme in liver cells responsible for breaking down acetaldehyde, which accumulates in the body and damages physical function. It is suspected to be carcinogenic and have serious health effects [39]. During the secretion season, the number of scented gland cells increases and secretes musk, so the downregulation of ALDH gene expression can promote the proliferation of scented gland cells and the synthesis of musk.

The genes c188759.graph_c0(3.1.6.12/*ARSP*) and c181507.graph_c0(HPSE2) in the glycosaminoglycan degradation (ko00531) was downregulated 2.317 and 1.831 times, respectively. Heparanase2 (HPSE2) can inhibit cell proliferation, invasion, and migration, inhibit EMT, and promote cell apoptosis [40]. During the secretion season, HPSE2 was downregulated, thereby promoting the proliferation of scented gland cells in large quantities.

Four genes were downregulated, and three genes were upregulated in glycolysis/glyconeogenesis (ko00010). The upregulation of adenosine diphosphate-dependent glucose antagonist RNA 1 (ADPGK-AS1) in the adenosine diphosphate-dependent glucose (ADPGK) family of genes increases in gastric cancer tissues and cell lines. Silencing ADPGK-AS1 can inhibit GC cell proliferation and migration [41]. Glyceraldehyde-3-phosphate dehydrogenase (GAPDH) is an essential key enzyme in the process of glycolysis, gluconeogenesis, and Calvin cycle metabolism. It is one of the most basic enzymes to maintain cell life activities and is a protein that can be highly expressed and widely exists in cells. Combined with downregulated genes, it can promote the proliferation of scented gland cells and the synthesis of musk during the muskrat secretion season.

In this work, the significant changes in the metabolic pathways of nine saccharide substances (starch, sucrose, galactose, fructose, mannose metabolism, amino sugar, nucleoside sugar, glycosaminoglycan, pentose) in the scented gland were observed, which is closely related to the distribution of these saccharides. Although the regulatory changes in saccharide metabolism in the scented gland ensure its metabolism and energy requirements during the secretion season, the specific regulatory mechanism of saccharide synthesis and metabolism on the growth, proliferation, and secretion of musk by muskrat scented gland cells is not yet clear. Therefore, further research is needed.

## 5. Conclusions

In summary, analyzing the saccharide metabolism signaling pathway in the muskrat scented gland will help elucidate the regulatory mechanism of the gland during the musk secretion season. An investigation of the saccharide profile and metabolic gene expression in scented glands was conducted, laying the foundation for further research on the specific mechanism of musk secretion. In addition, this study can also be used to improve the formula of muskrat diets. During feeding, the ratio of various plant-based and cereal-based feeds with high saccharide content can be adjusted according to the secretion season.

## Figures and Tables

**Table 1:** Saccharide substances screening results in blood serum compared to the secretion season and non-secretion season.

#ID	Compounds	log_2_FC	VIP	Regulated
MEDN1108	1,6-anhydro--D-glucose	0.512	0.705	unchanged
MEDN1231	1-O-vanillyl--D-glucose	0.182	1.737	unchanged
MEDP0216	D-Arabitol	0.136	0.073	unchanged
MEDP0224	D-Fructose	0.159	0.293	unchanged
MEDN0220	D-Glucose	−0.141	0.467	unchanged
MEDN0221	D-Mannose	0.350	0.909	unchanged
MEDN0224	D-Trehalose	−0.130	0.705	unchanged
MEDN1129	Erythrose	−0.960	1.009	unchanged
MEDP0504	Glucosamine	−0.079	0.136	unchanged
MEDN0229	Lactose	−0.130	0.705	unchanged
MEDN0230	Lactulose	−0.130	0.705	unchanged
MEDN0570	L-Erythrulose	−0.960	1.009	unchanged
MEDN0231	L-Fucose	−0.302	0.375	unchanged
MEDN0232	L-Rhamnose	−0.302	0.375	unchanged
MEDN0233	Maltose	−0.130	0.705	unchanged
MEDN0808	Myoinositol	0.350	0.909	unchanged
MEDN1011	D-Mannitol	2.015	1.227	unchanged
MEDN0213	D-Sorbitol	0.463	1.207	unchanged
MEDP0221	Ribitol	0.136	0.073	unchanged
MEDN0498	Ribulose-5-Phosphate	0.230	0.499	unchanged
MEDP0700	Methyl beta-D-galactopyranoside	0.072	0.116	unchanged

FC: fold change is a measure describing how much a quantity changes going from an initial to a final value; Log_2_FC: expresses the FC of multiple values; VIP: the VIP value of the OPLS-DA model.

**Table 2:** Saccharide substance screening results in blood serum and musk during the secretion season.

#ID	Compounds	log_2_FC	VIP	Regulated
MEDN1108	1,6-anhydro--D-glucose	−1.325	0.931	unchanged
MEDN1231	1-O-vanillyl--D-glucose	−0.201	0.367	unchanged
MEDP0216	D-Arabitol	−0.477	0.310	unchanged
MEDP0224	D-Fructose	2.082	0.690	unchanged
MEDN0220	D-Glucose	2.230	1.386	up
MEDN0221	D-Mannose	0.435	0.728	unchanged
MEDN0224	D-Trehalose	−2.053	1.198	down
MEDN1129	Erythrose	1.361	0.426	unchanged
MEDP0504	Glucosamine	−0.305	0.324	unchanged
MEDN0229	Lactose	−2.053	1.198	down
MEDN0230	Lactulose	1.361	0.426	down
MEDN0570	L-Erythrulose	−4.920	1.223	unchanged
MEDN0231	L-Fucose	−4.920	1.223	down
MEDN0232	L-Rhamnose	−2.053	1.198	down
MEDN0233	Maltose	0.435	0.728	unchanged
MEDN0808	Myoinositol	0.655	0.444	unchanged
MEDN1011	D-Mannitol	1.906	1.403	up
MEDN0213	D-Sorbitol	−0.477	0.310	unchanged
MEDP0221	Ribitol	−0.308	0.284	unchanged
MEDN0498	Ribulose-5-Phosphate	0.366	0.554	unchanged
MEDP0700	Methyl beta-D-galactopyranoside	−1.325	0.931	unchanged

FC: fold change is a measure describing how much a quantity changes going from an initial to a final value; Log_2_FC: expresses the FC of multiple values; VIP: the VIP value of the OPLS-DA model.

**Table 3:** Saccharide substance abundance in blood serum and musk.

#ID	Compounds	Secretion Season Blood Serum (^0^/_0000_)	Non-Secretion Season Blood Serum (^0^/_0000_)	Musk (^0^/_0000_)
MEDN1108	1,6-anhydro-D-glucose	0.5026	0.7168	0.2860
MEDN1231	1-O-vanillyl-D-glucose	0.1600	0.1815	0.1579
MEDP0216	D-Arabitol	0.2314	0.2543	0.1827
MEDP0224	D-Fructose	90.20	100.7	77.74
MEDN0220	D-Glucose *	0.7925	0.7188	3.372
MEDN0221	D-Mannose	1.037	1.322	1.787
MEDN0224	D-Trehalose *	5.223	4.772	1.150
MEDN1129	Erythrose	0.6394	0.3287	0.8446
MEDP0504	Glucosamine	18.43	17.46	14.13
MEDN0229	Lactose *	5.223	4.772	1.150
MEDN0230	Lactulose *	5.223	4.772	1.150
MEDN0570	L-Erythrulose	0.6394	0.3287	0.8446
MEDN0231	L-Fucose *	1.003	0.8141	0.0269
MEDN0232	L-Rhamnose	1.003	0.8141	0.0269
MEDN0233	Maltose	5.223	4.772	1.150
MEDN0808	Myoinositol	1.037	1.322	1.787
MEDN1011	D-Mannitol	0.0460	0.1858	0.2927
MEDN0213	D-Sorbitol *	0.2093	0.2885	1.081
MEDP0221	Ribitol	0.2314	0.2543	0.1827
MEDN0498	Ribulose-5-Phosphate	1.017	1.192	0.9.633
MEDP0700	Methyl beta-D-galactopyranoside	1.284	1.349	1.739

The third column to the end indicates the normalized metabolite abundance for each sample. * indicates saccharides with significant changes.

**Table 4:** Changes in the expression of saccharide metabolism-related genes in muskrat scented gland tissues in secretion and non-secretion seasons.

Unigene ID	Log_2_FC	Regulated
**A. Starch and sucrose metabolism (ko00050)-related genes.**		
c186238.graph_c1(2.7.1.1/*HK*)K00844	−2.487	down
c176194.graph_c0(2.4.1.18/*GBE1*)K00700	−2.236	down
c184127.graph_c0(2.4.1.1/*PYG*)K00688	−1.826	down
c156542.graph_c0(2.4.1.17/*UGT*)K00699	−1.834	down
**B. Galactose metabolism (ko00052)-related genes.**		
c186238.graph_c1(2.7.1.1/*HK*)K00844	−2.487	down
c167477.graph_c0(1.1.1.21/*AKR1B*)K00011	2.293	up
**C. Fructose and mannose metabolism (ko00051)-related genes.**		
c186238.graph_c1(2.7.1.1/*HK*)K00844	−2.487	down
c167477.graph_c0(1.1.1.21/*AKR1B*)K00011	2.293	up
c189136.graph_c1(5.4.2.8/*manB*)K01840	1.677	up
**D. Pentose phosphate pathway (ko00030)-related genes.**		
c186752.graph_c0(1.1.1.44/*PGD*)K00033	2.004	up
c179458.graph_c0(5.3.1.6/*rpiA*)K01807	1.379	up
**E. Amino sugar and nucleotide sugar metabolism (ko00520)-related genes.**		
c186238.graph_c1(2.7.1.1/*HK*)K00844	−2.487	down
c189136.graph_c1(5.4.2.8/*manB*)K01840	1.677	up
c155840.graph_c0(3.2.1.14/*E3.2.1.14*)K01183	−2.646	down
c188901.graph_c1(1.14.18.2/*CMAH*)K08080	−1.675	down
**F. Glycosaminoglycan degradation (ko00531)-related genes.**		
c188759.graph_c0(3.1.6.12/*ARSP*)K01135	−2.317	down
c181507.graph_c0(*HPSE2*)K07965	−1.831	down
**G. Pentose and glucuronate interconversions (ko00040)-related genes.**		
c156542.graph_c0(2.4.1.17/*UGT*)K00699	−1.834	down
c167477.graph_c0(1.1.1.21/*AKR1B*)K00011	2.293	up
c166519.graph_c0(1.2.1.3/*ALDH2*)K00128	−1.51	down
c188162.graph_c0(1.2.1.3/*ALDH1*)K00128	−1.632	down
c184675.graph_c1(1.2.1.3/*ALDH4*)K00128	3.62	up
**H. Glycolysis/gluconeogenesis (ko00010)-related genes.**		
c186238.graph_c1(2.7.1.1/*HK*)K00844	−2.487	down
c157360.graph_c0(2.7.1.147/*ADPGK*)K08074	1.421	up
c186915.graph_c0(1.2.1.12/*GAPDHa*)K00134	−7.118	down
c178269.graph_c0(1.2.1.12/*GAPDH*)K00134	2.329	up
c166519.graph_c0(1.2.1.3/*ALDH2*)K00128	−1.51	down
c184675.graph_c1(1.2.1.3/*ALDH4*)K00128	3.62	up
c188162.graph_c0(1.2.1.3/*ALDH1*)K00128	−1.632	down

FC: fold change is a measure describing how much a quantity changes going from an initial to a final value; Log_2_FC: expresses the FC of multiple values.

## Data Availability

The raw data supporting the conclusion of this work will be made available by the authors without undue reservation.

## Supplementary Materials

The following supporting information can be downloaded at: https://www.mdpi.com/article/10.3390/ani14243705/s1, Figure S1: The metabolism of starch and sucrose, Figure S2: The metabolism of galactose, Figure S3: The metabolism of fructose and mannose, Figure S4: The pathway of pentose phosphate, Figure S5: The metabolism of amino sugar and nucleotide sugar, Figure S6: The degradation of glycosaminoglycan, Figure S7: The interconversions between pentose and gluconate, Figure S8: The interconversions between glycolysis and glyconeogenesis.
