# Supplementary material for: An Investigation of the Saccharides Profile and Metabolic Gene Expression in Muskrat Scented Glands in Different Secretion Seasons
Source: Animals (Basel). 2024 Dec 22;14(24):3705. doi: 10.3390/ani14243705 (PMC11672420; doi:10.3390/ani14243705)
Supplement: Supplementary file 1 [file animals-14-03705-s001.zip › animals-3365497-supplementary.pdf]

Supplement information

Investigation the Saccharides Profile and Metabolic Gene Expression in Muskrat Scented Glands in Different Secretion Season

Juntong Zhou, Defu Hu *, Nuannuan Feng, Shuqiang Liu and Junqing Li

Department of Ecology, School of Ecology and Nature Conservation, Beijing Forestry University, Beijing 100083, China; zhoujuntong908020@126.com (J.Z.); Fnn1990123@163.com (N.F.); shuqiangliu@163.com (S.L.); lijq@bjfu.edu.cn (J.L.)

* Correspondence: hudf@bjfu.edu.cn

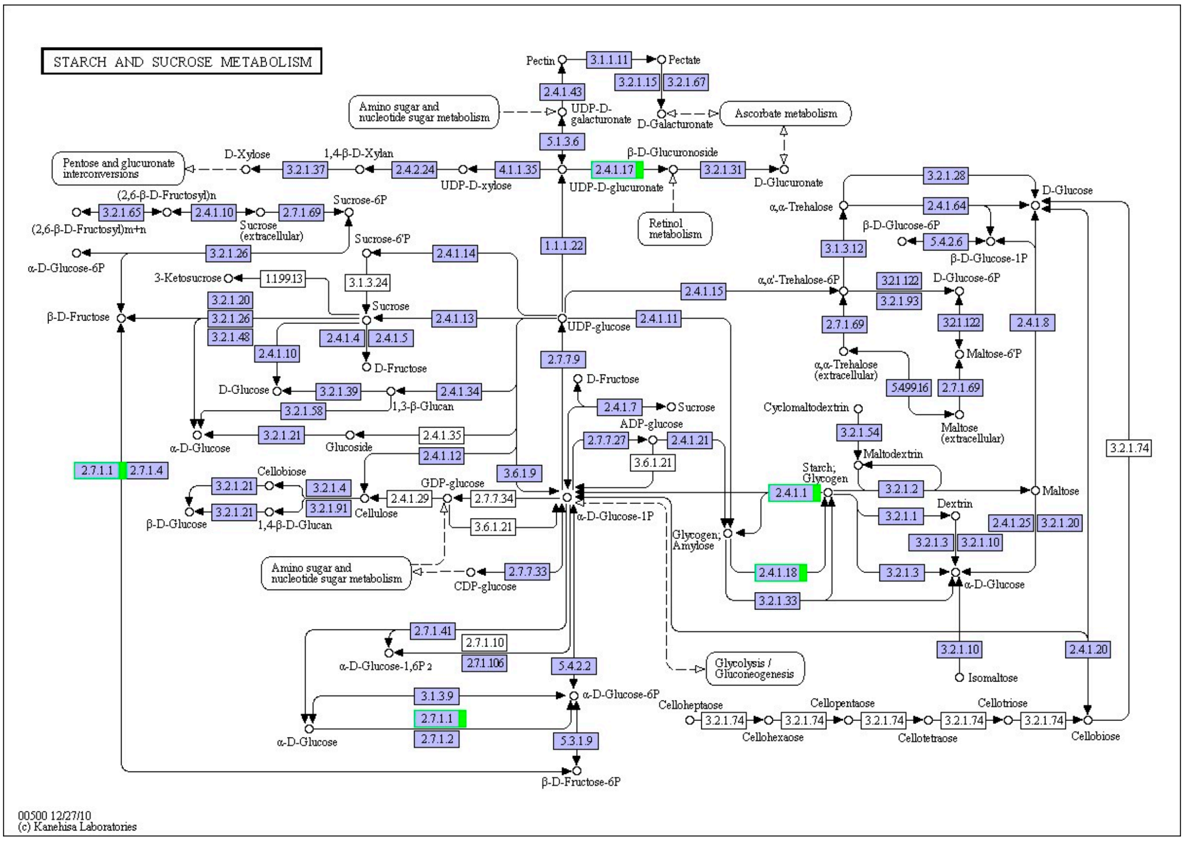

Figure S1. The metabolism of starch and sucrose.

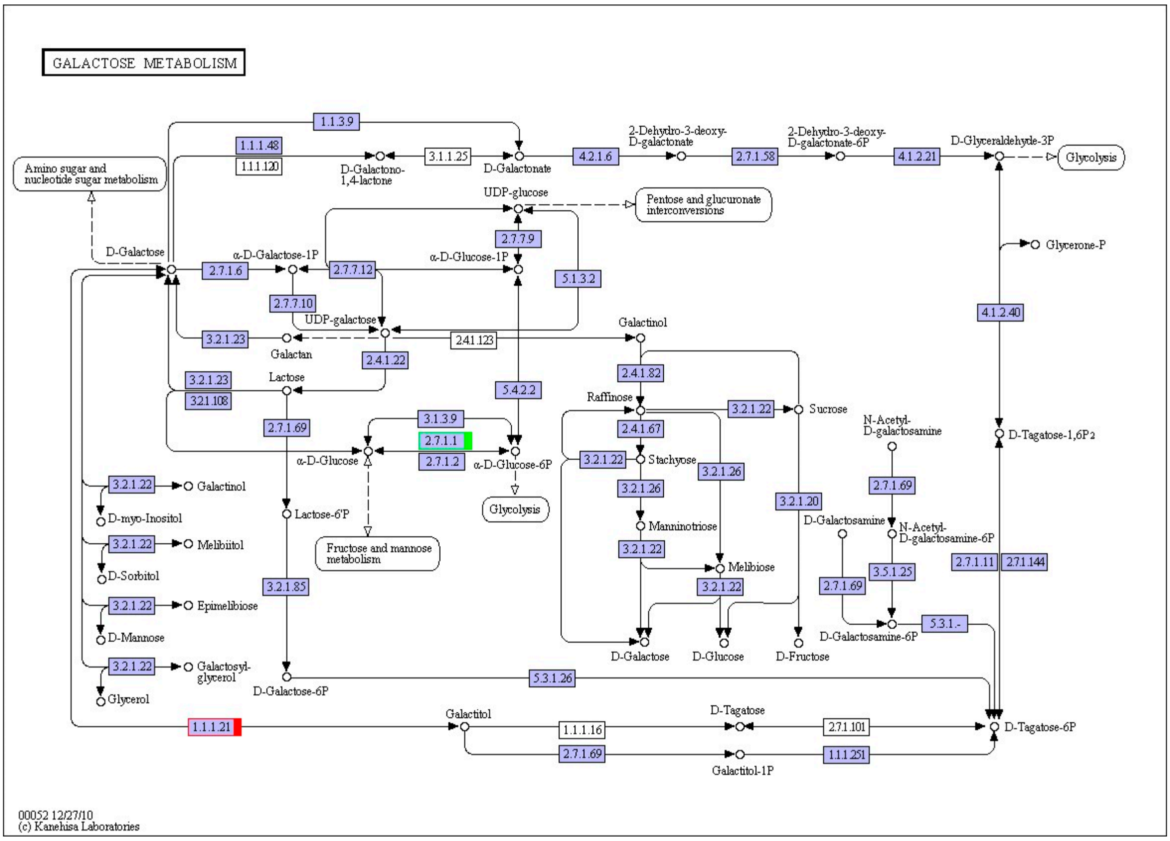

Figure S2. The metabolism of galactose.

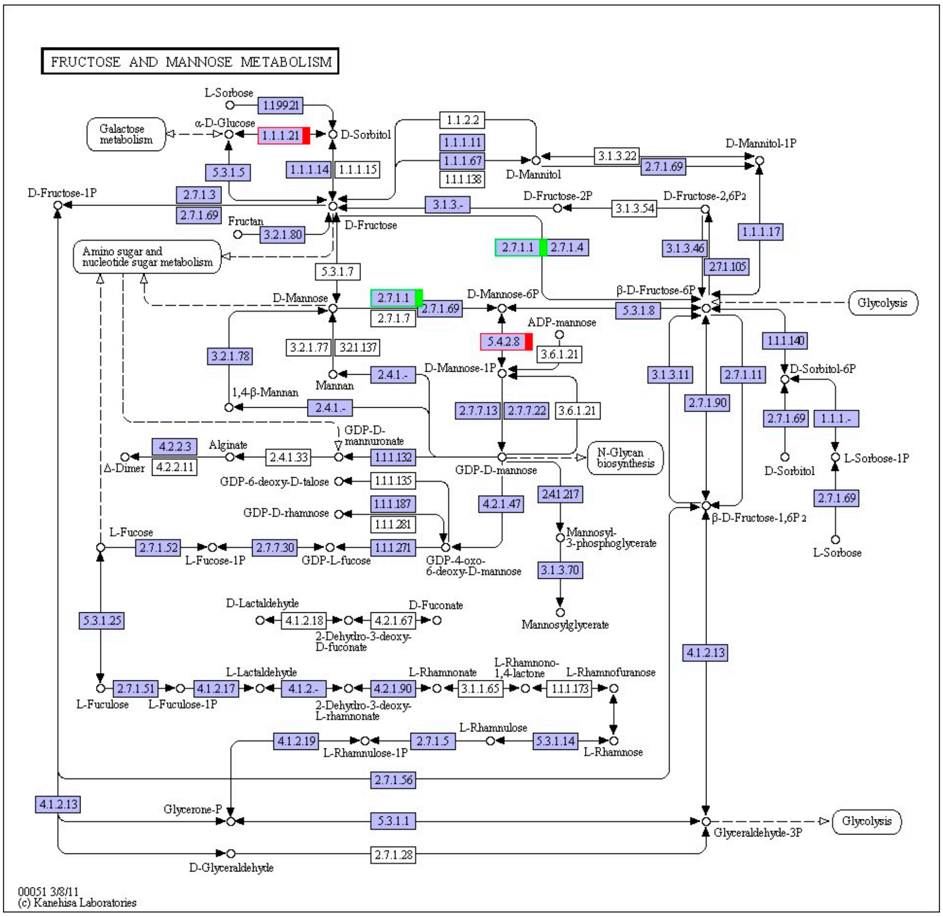

Figure S3. The metabolism of fructose and mannose.

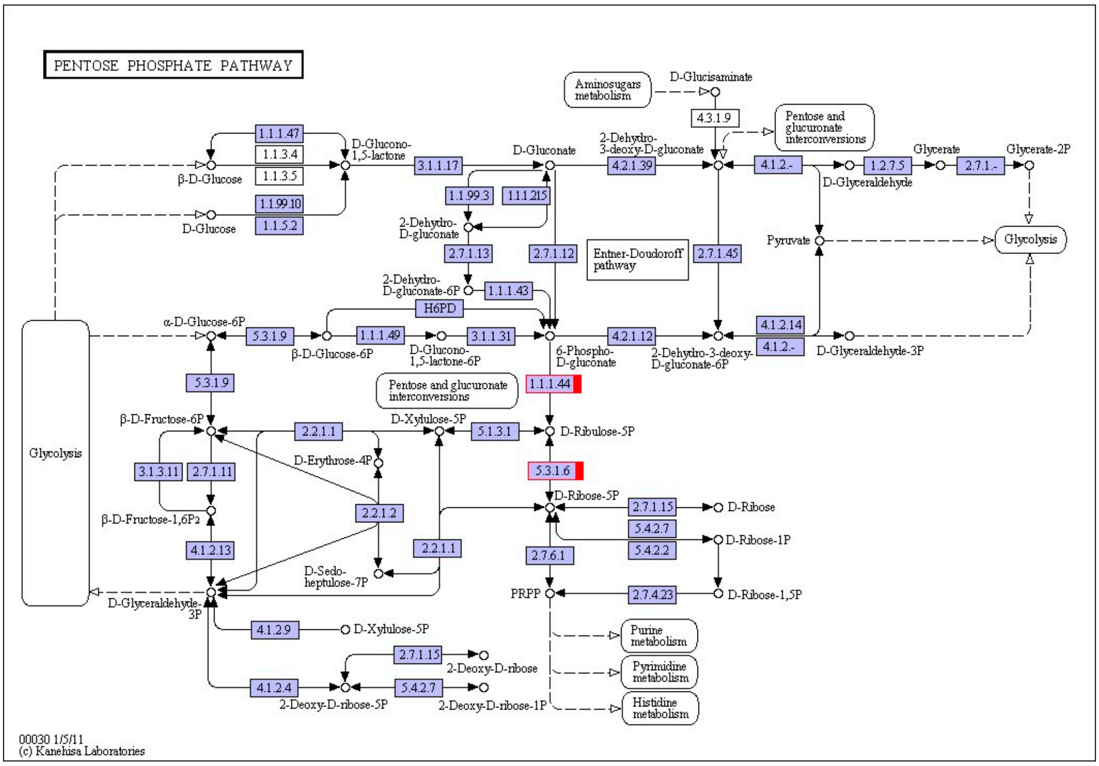

Figure S4. The pathway of pentose phosphate.

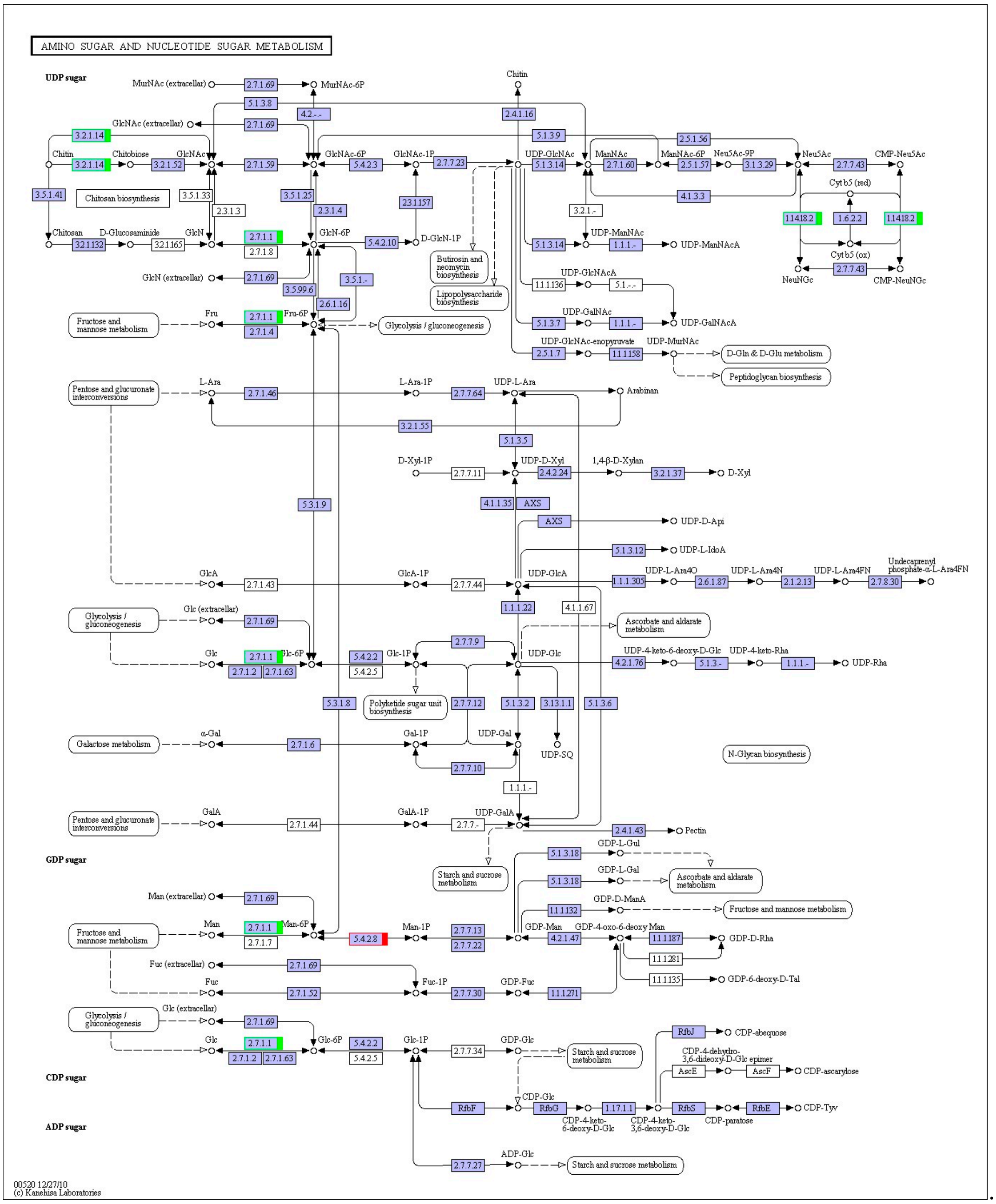

Figure S5. The metabolism of amino sugar and nucleotide sugar.

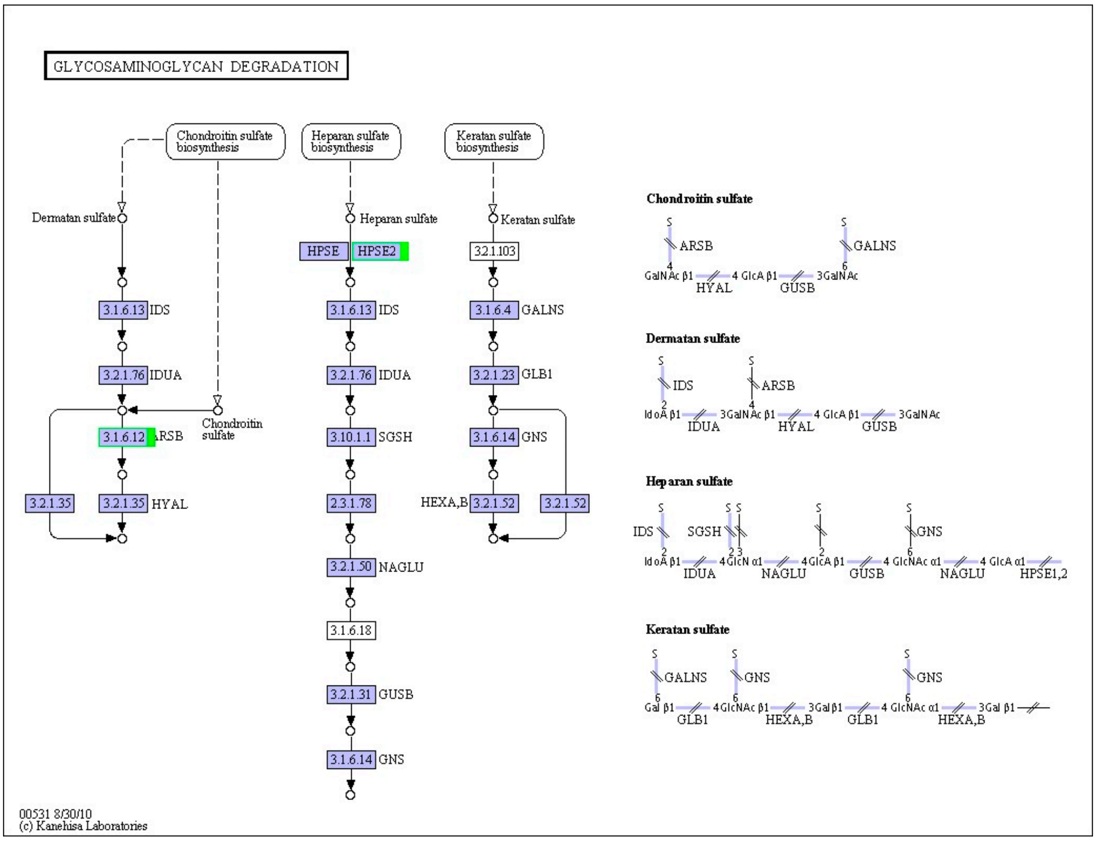

Figure S6. The degradation of glycosaminoglycan.

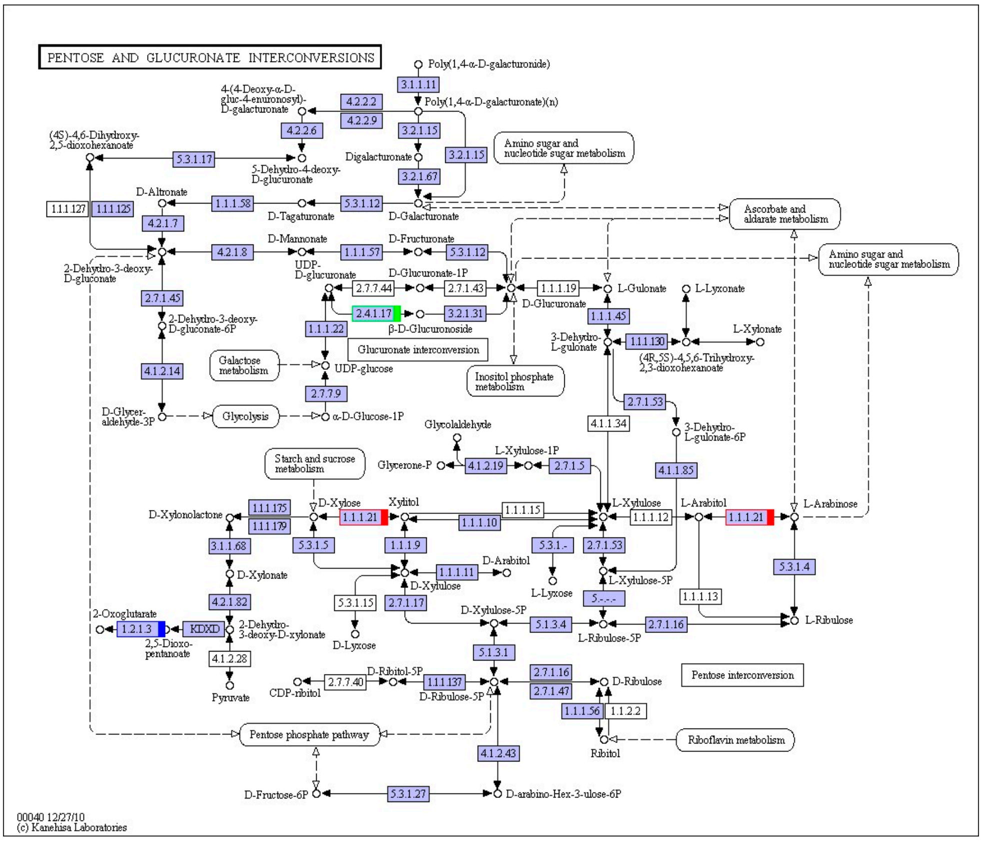

Figure S7. The interconversions between pentose and gluconate.

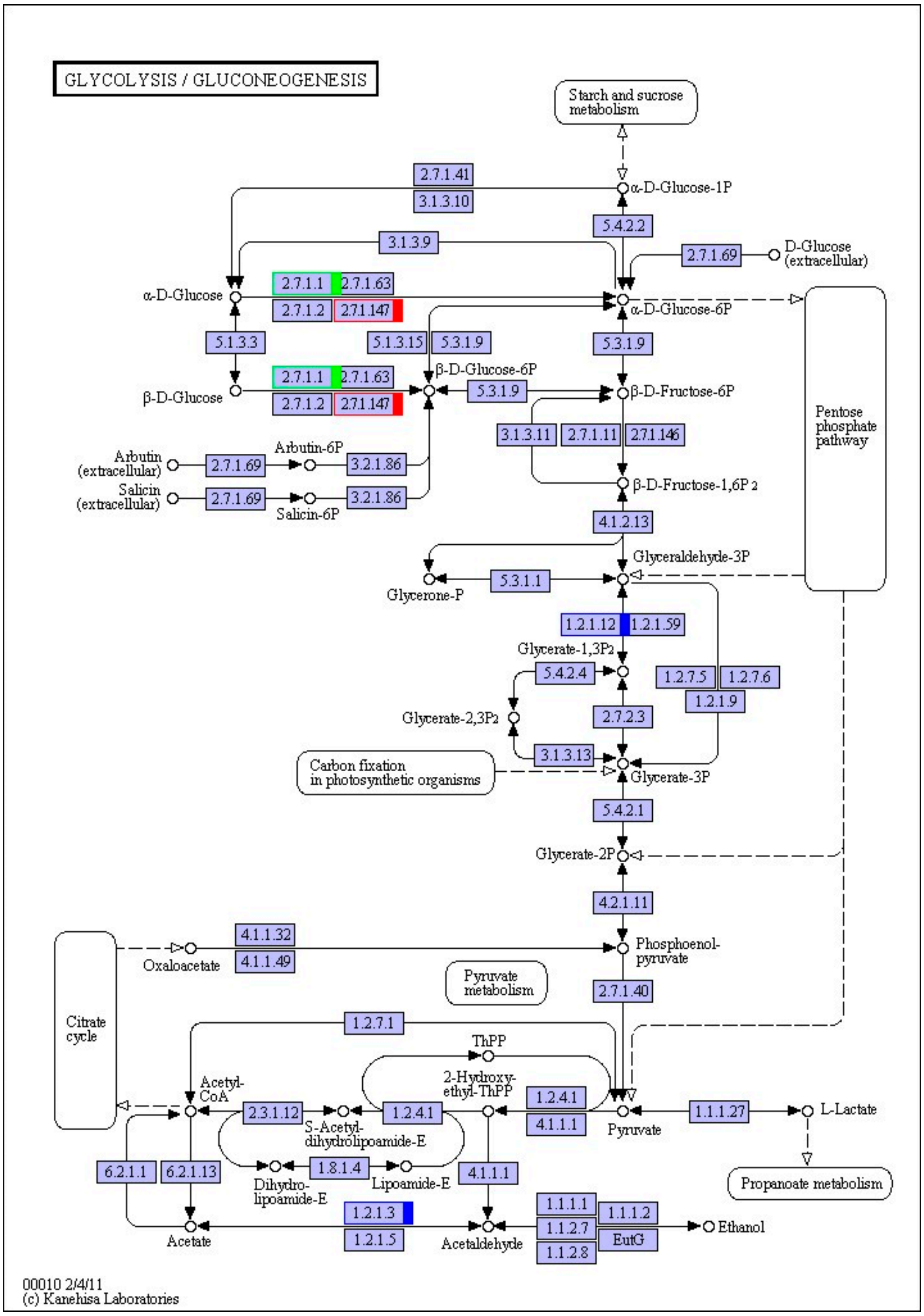

Figure S8. The interconversions between glycolysis and glyconeogenesis.
